# O5.2. PREDICTORS OF CARDIOMETABOLIC RISK IN THE YEAR AFTER ONSET OF PSYCHOSIS: A PROSPECTIVE COHORT STUDY

**DOI:** 10.1093/schbul/sby015.216

**Published:** 2018-04-01

**Authors:** Fiona Gaughran, John Lally, Khalida Ismail, David Hopkins, Shubulade Smith, Kathryn Greenwood, Poonam Sood, Brendon Stubbs, Dominic Stringer, Daniel Stahl, Anthony David, Robin Murray, Anita Patel

**Affiliations:** 1 King’s College London; 2 Institute of Psychiatry, Psychology & Neuroscience, King’s College London; 3 University of Sussex; 4 Queen Mary, University of London

## Abstract

**Background:**

The first episode of psychosis (FEP) is a critical time to prevent the onset of weight gain, cardiovascular and metabolic disease. However, little is known about the influence of patient characteristics and lifestyle on these outcomes

**Methods:**

We conducted a prospective cohort study over 12 months of 294 people with FEP investigating the influence of lifestyle factors and medication on cardiometabolic outcomes over 12 months. Information on sociodemographics, lifestyle (physical activity (PA), sedentary behaviour (SB) nutrition, smoking), medication and service use and mental health symptoms was collected at baseline and after twelve months.

**Results:**

There were high rates of cardiometabolic abnormalities and unhealthy lifestyle choices on first presentation with psychosis, increasing over the subsequent 12 months. Obesity rates rose from 17.8% to 23.7% while the proportion with Hba1c levels >/= 39mmol/mol rose from 12% to 23.7%. White participants were more at risk of developing central obesity while there were highly clinically relevant increases in mean Hba1c in those of non-white ethnicity from 36.4 to 39.7mmol/mol. We found no association between lifestyle or medication with either baseline or 12-month cardiometabolic outcomes.

**Discussion:**

Cardiometabolic risk factors and unhealthy lifestyle behavior are already prevalent in those with early psychosis and worsen in the year following first presentation, making this an important time for preventative strategies. We found no evidence however that such strategies should be preferentially directed towards those reporting less healthy lifestyle habits. Patterns of emergence of cardiometabolic risk over the first year of psychosis varied by ethnicity.

